# Chemerin-9 Attenuates Experimental Abdominal Aortic Aneurysm Formation in ApoE^−/−^ Mice

**DOI:** 10.1155/2021/6629204

**Published:** 2021-04-17

**Authors:** Shuxiao Chen, Chenglin Han, Shuai Bian, Jianfeng Chen, Xuedong Feng, Gang Li, Xuejun Wu

**Affiliations:** ^1^Department of Vascular Surgery, Shandong Provincial Hospital, Cheeloo College of Medicine, Shandong University, Jinan, Shandong, China; ^2^Department of Urology, Shandong Provincial Hospital, Cheeloo College of Medicine, Shandong University, Jinan, Shandong, China; ^3^Department of Vascular Surgery, Shandong Provincial Hospital Affiliated to Shandong First Medical University, Jinan, Shandong, China

## Abstract

Chronic inflammation plays an essential role in the pathogenesis of abdominal aortic aneurysm (AAA), a progressive segmental abdominal aortic dilation. Chemerin, a multifunctional adipocytokine, is mainly generated in the liver and adipose tissue. The combination of chemerin and chemokine-like receptor 1 (CMKLR1) has been demonstrated to promote the progression of atherosclerosis, arthritis diseases, and Crohn's disease. However, chemerin-9 acts as an analog of chemerin to exert an anti-inflammatory effect by binding to CMKLR1. Here, we first demonstrated that AAA exhibited higher levels of chemerin and CMKLR1 expression compared with the normal aortic tissues. Hence, we hypothesized that the chemerin/CMKLR1 axis might be involved in AAA progression. Moreover, we found that chemerin-9 treatment markedly suppressed inflammatory cell infiltration, neovascularization, and matrix metalloproteinase (MMP) expression, while increasing the elastic fibers and smooth muscle cells (SMCs) in Ang II-induced AAA in ApoE^−/−^ mice. This demonstrated that chemerin-9 could inhibit AAA formation. Collectively, our findings indicate a potential mechanism underlying AAA progression and suggest that chemerin-9 can be used therapeutically.

## 1. Introduction

Abdominal aortic aneurysm (AAA), a typically asymptomatic dilatation of the abdominal aorta, is always diagnosed incidentally [1]. The risk of aneurysm rupture is notably elevated with increasing abdominal aortic diameters [2, 3]. Currently, surgical intervention through open or endovascular repair is recommended for advanced AAA patients with a maximal aortic diameter of >5 cm; however, no drugs effectively limit the growth of small aneurysms [4–6]. Therefore, the discovery of novel, efficient drugs that inhibit AAA progression has immense clinical significance.

The pathological characteristics of AAA include chronic inflammation, vascular smooth muscle cell (VSMC) apoptosis, and extracellular matrix (ECM) destruction [7]. To our knowledge, chronic inflammation plays an essential role in early events involving AAA initiation and progression [8]. Inflammatory cell infiltration in the media and adventitia contributes to the accumulation of various proinflammatory mediators, such as interleukin-1 (IL-1*β*), tumor necrosis factor (TNF-*α*), interleukin-6 (IL-6), and matrix metalloproteinases (MMPs) [9, 10], further resulting in pathological remodeling of the aortic wall though degradation of the ECM [11, 12]. Targeting these inflammatory mechanisms may represent a promising strategy for treating AAA.

Chemerin, a multifunctional adipokine encoded by tazarotene-induced gene 2 (TIG2), is excreted by the liver, platelets, white fat, and perivascular adipose tissue (PVAT). Chemokine-like receptor 1 (CMKLR1) is a G protein-coupled receptor that is mainly expressed in adipocytes, VSMCs, endothelial cells, macrophages, and natural killer cells [13]. Previous reports demonstrated that chemerin binding to CMKLR1 was involved in atherosclerosis, rheumatoid arthritis, and inflammatory bowel disease (IBD) by mediating inflammatory responses [14–21]. Therefore, we speculated that the levels of chemerin and CMKLR1 may be associated with AAA progression.

Chemerin-9 (149–157), comprising nine amino acids from the C-terminus of chemerin, retains most agonist activity [22]. In vivo, chemerin-9 exerts an anti-inflammatory effect by targeting CMKLR1, thus restraining the development of several diseases, including atherosclerosis, Alzheimer's disease, and pancreatogenic diabetes [22–24]. However, the role of chemerin-9 in AAA behavior has not yet been reported.

Here, we evaluated the levels of chemerin and CMKLR1 in AAA and established their interactions with chemerin-9. We also demonstrated that chemerin-9 effectively inhibited the development of angiotensin II (Ang II)-induced AAA in ApoE^−/−^ mice.

## 2. Materials and Methods

### 2.1. Clinical Specimen Collection

This study was approved by the Human Research Committee of Shandong Provincial Hospital affiliated with Shandong University, and the procedures conformed to the ethical guidelines of the Declaration of Helsinki. Informed consent was obtained for experiments with human subjects. All patients with AAA were diagnosed using computerized tomography (CT) and magnetic resonance imaging (MRI).

Human blood samples were collected from patients with AAA who were admitted to Shandong Provincial Hospital and ten age-matched healthy volunteers. Human AAA specimens were obtained from patients undergoing surgical AAA repair at the Department of Vascular Surgery of Shandong Provincial Hospital. As a control, six age-matched normal human aorta samples were obtained from multiorgan donors. Patients with aortitis, connective tissue disorders, or ruptured aneurysms were excluded.

### 2.2. Experimental Animals

Eight-week-old male ApoE^−/−^ mice (*n* = 60) were purchased from Beijing Charles River Company and housed at the Animal Centre of Shandong Provincial Hospital. The animals were kept on a 12 h dark/light cycle with free access to water and a high-fat diet (containing 1.5% cholesterol). All experimental procedures performed on ApoE^−/−^ mice were approved by the Animal Care and Use Committee of Shandong Provincial Hospital and were conducted according to institutional guidelines and the Guide for the Care and Use of Laboratory Animals by the National Institutes of Health (1996).

### 2.3. Animal Model and Experimental Design

Sixty ApoE^−/−^ mice were randomly divided into Sham, AAA, and chemerin-9 groups (*n* = 20 per group). Mice in the Sham group were infused with saline (1.44 mg/kg/d); those in the AAA group, with Ang II (1.44 mg/kg/d, GL Biochem); and those in the chemerin-9 group, with Ang II (1.44 mg/kg/d) and chemerin-9 (7.7 *μ*g/kg/h; GenScript). Treatments were administered by subcutaneously implanted micro-osmotic pumps (Alzet Model 2004, Durect, Cupertino, USA) in the dorsum of the neck for 28 days. The appropriate doses of chemerin-9 were selected based on their inhibitory effects on atherosclerosis progression [23]. At the indicated time points, mice were sacrificed for image acquisition and tissue harvesting.

### 2.4. Measurement of Aortic Diameter

A B-ultrasound system (Vevo 2100, Visual Sonics Inc.) was used to examine the maximum inner luminal diameters of the abdominal aortas at 0, 7, 14, and 28 days after pump implantation. Two researchers blinded to the group assignment independently measured all the abdominal aortic diameters.

### 2.5. Enzyme-Linked Immunosorbent Assay

Blood samples were allowed to clot at room temperature for 2 h and centrifuged at 2000 g for 20 min. The sera were collected and stored in aliquots at −80°C until further use. Total chemerin levels in serum (1 : 5 dilution) were determined using commercially available human and mouse chemerin ELISA kits (Jonln, JL47214, and JL20146, China) according to the manufacturer's instructions.

### 2.6. Real-Time Quantitative Polymerase Chain Reaction (qRT-PCR) Analysis

Total RNA was extracted from the aortas and reverse transcribed to cDNA using the PrimeScript RT reagent kit with gDNA Eraser (RR047, Takara, Japan). qRT-PCR was performed according to the manufacturer's protocol to detect the mRNA levels of chemerin, CMKLR1, MMP-2, MMP-9, and GAPDH in murine samples using SYBR Premix Ex Taq II (RR820, Takara, Japan). The relative expression levels were calculated using the following equation: relative mRNA level = 2^− (Δ*Ct* sample^ ^−^ ^Δ*Ct* control)^. All experiments were performed at least three times. The primers used in the PCR are listed in Table 1.

### 2.7. Western Blot

Total proteins were extracted from the aortic segments using a lysis buffer (Beyotime, China). These lysates were then separated by 10% sodium dodecyl sulfate-polyacrylamide gel electrophoresis (SDS-PAGE, Invitrogen, USA), transferred onto polyvinylidene fluoride (PVDF) membranes (Millipore, USA), and then incubated with primary antibodies against chemerin (1 : 1000, ab103153, Abcam, USA), CMKLR1 (1 : 1000, AP06779, Origene, USA), MMP-2 (1 : 1000, ab86607, Abcam, USA), MMP-9 (1 : 1000, ab38898, Abcam, USA), and GAPDH (1 : 10000, ab8245, Abcam, USA) overnight at 4°C. The next day, the blots were incubated with horseradish peroxidase- (HRP-) conjugated AffiniPure goat anti-rabbit IgG (1 : 5000, SA00001-2, Proteintech, China) or anti-mouse IgG (1 : 5000, SA00001-1, Proteintech, China) for 1h at room temperature. Proteins were visualized using Amersham Imager (Cytiva, USA) and quantified using ImageJ software.

### 2.8. Histopathology

The dissected abdominal aortas were fixed in 4% paraformaldehyde and embedded in Tissue-Tek O.C.T. Compound (Sakura Finetek) to prepare cryostat sections. The embedded tissue was cut into 4 *μ*m thick serial sections and fixed with ice-cold acetone. Briefly, the slides were stained with hematoxylin and eosin (HE) solution (Servicebio, G1005, China) and Elastica van Gieson (EVG) kit (Servicebio, G1042, China), according to the respective standard protocols. Morphological changes in the abdominal aorta were observed using HE staining. The degradation of elastic fibers was observed using EVG staining. Images were obtained using a confocal laser scanning microscope (Leica, Solms, Germany).

Elastin degradation scores were evaluated as previously described (1, no elastin degradation or mild elastin degradation; 2, moderate; 3, moderate to severe; and 4, severe elastin degradation) [25].

### 2.9. Immunofluorescence

The localizations and expression of chemerin and CMKLR1 were compared using immunofluorescence (IF) staining. Tissues were stained for 2 h at room temperature in the presence of anti-chemerin (1 : 200, ab72965, Abcam, USA) and anti-CMKLR1 (1 : 200, AP06779, Origene, USA) antibodies, followed by staining for 1 h at room temperature with appropriate Alexa Fluor 488/647-conjugated secondary antibodies (Invitrogen, Carlsbad, CA, USA). Tissue sections were stained with DAPI for 5 min before imaging.

### 2.10. Immunohistochemistry

The VSMCs, macrophages, B cells, T cells, neovascularization, and MMPs in the abdominal aorta were visualized by staining with anti-*α*- smooth muscle cell (1 : 200, ab5694, Abcam, USA), anti-CD68 (1 : 200, ab125212; Abcam, USA), anti-B220 (1 : 200; ab64110; Abcam, USA), anti-CD8 (1 : 200, ab22378; Abcam, USA), anti-CD31 (1 : 200, ab28364; Abcam, USA), anti-matrix metalloproteinase (MMP)-2 (1 : 200, ab86607, Abcam, USA), and anti-MMP-9 (1 : 200, ab38898, Abcam, USA) antibodies.

Cryostat sections were incubated with the primary antibodies at 37°C for 1 h and the second antibody at room temperature for 30 min. The sections were then incubated with biotin at room temperature for 30 min. Immune complexes were visualized using the AEC Peroxidase Substrate Kit (Solarbio, China). The slides were counterstained with hematoxylin. Images were captured using confocal laser scanning microscopy (Leica, Solms, Germany) and analyzed by Image-Pro Plus software.

### 2.11. Statistical Analysis

All data are expressed as mean ± standard deviation (SD). All statistical analyses were performed using GraphPad Prism 8 (GraphPad Software, Inc.). Statistical differences were evaluated by Student's *t*-test for two groups and one-way analysis of variance (ANOVA) followed by the Newman-Keuls test for multigroup data analysis. Statistical significance was set at *P* < 0.05.

## 3. Results

### 3.1. Chemerin and CMKLR1 Were Upregulated in Human AAA

**Figure 1:**
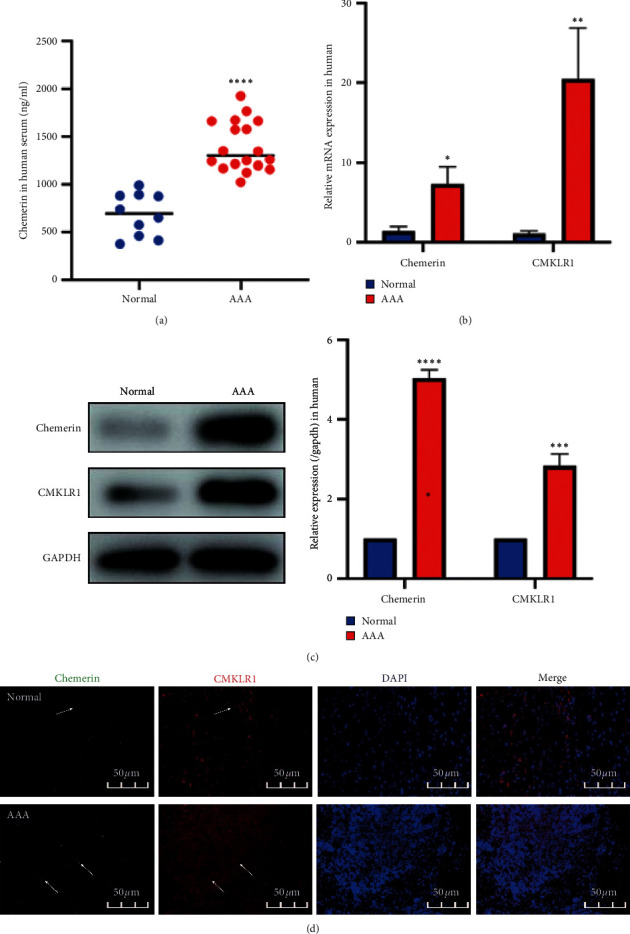
The expression of chemerin and CMKLR1 in humans. (a) Changes in circulating chemerin levels in humans. (b) Relative mRNA expression of chemerin and CMKLR1 in the human aortas. (c) Representative western blot images (left) and semiquantitative analysis (right) of protein expression of chemerin and CMKLR1 in the human aortas. (d) Double immunofluorescence staining for chemerin and CMKLR1 in human aortas. The data are shown as mean ± SD. ^*∗*^*P* < 0.05, ^*∗∗*^*P* < 0.01, ^*∗∗∗*^*P* < 0.001, ^*∗∗∗∗*^*P* < 0.0001 vs. the normal group.

We first examined whether there was difference in chemerin expression between patients with AAA and healthy individuals using ELISA. The data in Figure 1(a) show that the chemerin levels in the AAA group were significantly higher than those in the normal group. Moreover, qRT-PCR and western blot results showed that the levels of chemerin and CMKLR1 in human AAA tissues were also remarkably elevated (Figures 1(b)–1(c)). IF staining was performed to investigate the expression of chemerin and CMKLR1. Chemerin was located in the adventitia binding to CMKLR1, and the expression of chemerin and CMKLR1 in AAA was upregulated compared with that in normal aortic tissue (Figure 1(d)).

### 3.2. Chemerin and CMKLR1 Were Elevated in Experimental AAA

**Figure 2:**
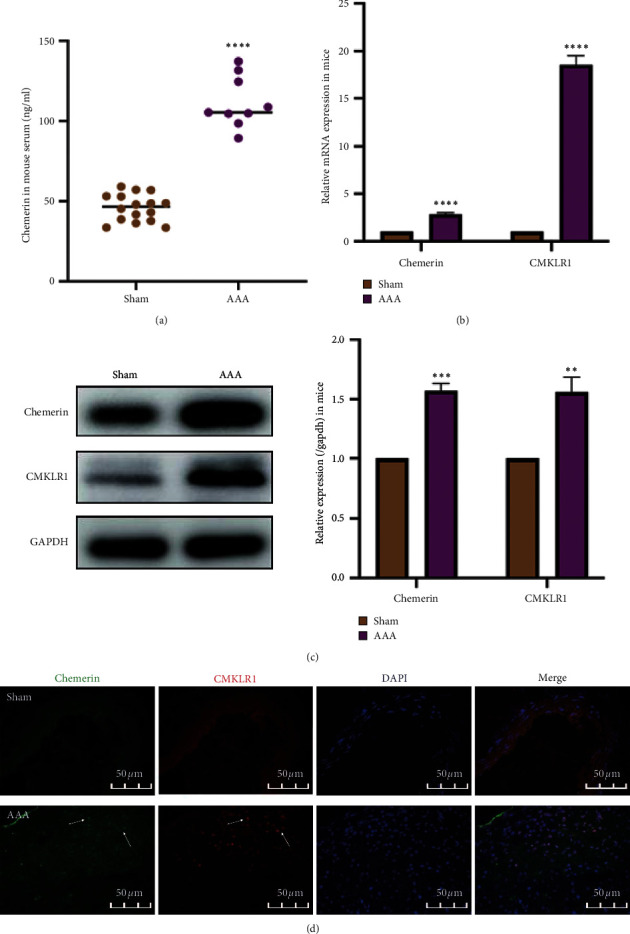
The expression of chemerin and CMKLR1 in mice. (a) Changes in circulating chemerin levels in mice (*n* = 16/Sham group; *n* = 9/AAA group). (b) Relative mRNA expression of chemerin and CMKLR1 in the mice aortas (*n* = 6/group). (c) Representative western blot images (left) and semiquantitative analysis (right) of protein expression of chemerin and CMKLR1 in the mice aortas (*n* = 6/group). (d) Double immunofluorescence staining for chemerin and CMKLR1 in the mice aortas. The data are shown as mean ± SD. ^*∗∗*^*P* < 0.01, ^*∗∗∗*^*P* < 0.001, ^*∗∗∗∗*^*P* < 0.0001 vs. the Sham group.

Subsequently, we measured the levels of chemerin and CMKLR1 in the experimental AAA model. ELISA demonstrated increased expression of chemerin in the serum of AAA mice (Figure 2(a)), and the AAA group exhibited higher levels of chemerin and CMKLR1 compared with the Sham group, as shown in Figures 2(b) and 2(c). Similar results were also obtained using IF staining (Figure 2(d)). Taken together, these data suggested that higher chemerin and CMKLR1 expression may be implicated in AAA progression.

### 3.3. Chemerin-9 Limited the Enlargement of the Abdominal Aorta

**Figure 3:**
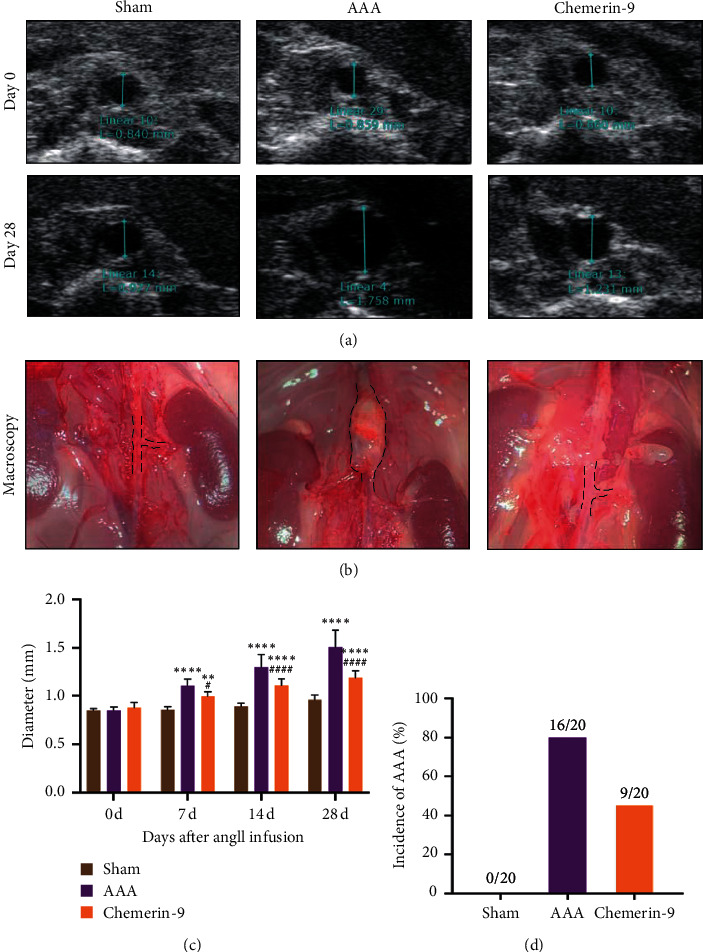
Effects of chemerin-9 on abdominal aortic diameter in ApoE^−/−^ mice. (a) Representative ultrasonography images on the 0 and 28 days after pump implantation (*n* = 20/group). (b) Representative microscopical images of the abdominal aorta in mice on the 28th day. (c) The maximal diameters of each group on the 0, 7, 14, and 28 days after pump implantation (*n* = 20/group). (d) The AAA incidence of each group 28 days after pump implantation (*n* = 20/group). The data are shown as mean ± SD. ^*∗∗*^*P* < 0.01, ^*∗∗∗∗*^*P* < 0.0001 vs. the Sham group; ^#^*P* < 0.05, ^####^*P* < 0.0001 vs. the AAA group.

We next explored the role of chemerin analog, chemerin-9, in AAA formation. Ang II and chemerin-9 were infused into ApoE^−/−^ mice via micro-osmotic pumps for 28 days. B-ultrasound was performed at the indicated time points to determine the effect of model building and the variation of lumen diameter. The results indicated that, with a prolonged period of Ang II infusion, the lumen diameter gradually increased; simultaneously, chemerin-9 remarkably suppressed the enlargement of abdominal aorta (Figures 3(a)–3(c)). The incidence of AAA in Sham, AAA, and chemerin-9 groups was 0%, 80%, and 45%, respectively (Figure 3(d)), indicating that chemerin-9 could prevent AAA formation to a certain degree.

### 3.4. Chemerin-9 Repaired the Histopathological Lesion of the Aneurysm Wall

**Figure 4:**
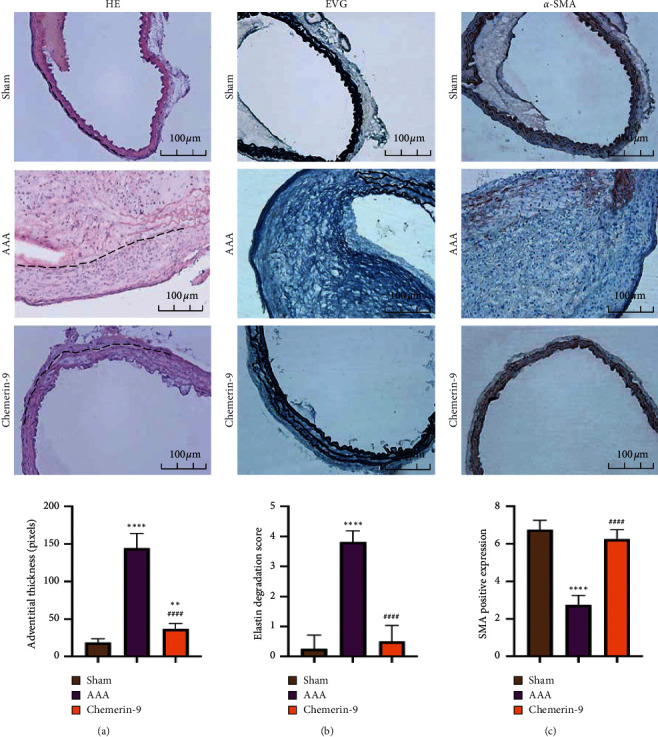
Effects of chemerin-9 on the morphological changes of the abdominal aorta in ApoE^−/−^ mice. (a) Histopathological analysis of representative abdominal aortas of the Sham group, AAA group and chemerin-9 group using HE staining (upper) and quantification (lower) of adventitia thickness (*n* = 6/group). The adventitial area is shown with black dotted lines. (b) Representative EVG staining images (upper) of elastin and semiquantitative analysis (lower) of elastic laminae deficiency in the medial layer of the aneurysm (*n* = 6/group). (c) Representative IHC staining images (upper) of SMCs and semiquantitative analysis (lower) of depleted medial smooth muscle in three groups (*n* = 6/group). The data are shown as mean ± SD. ^*∗∗*^*P* < 0.01; ^*∗∗∗∗*^*P* < 0.0001 vs. the Sham group; ^####^*P* < 0.0001 vs. the AAA group.

Given that the destruction of media elasticity and the apoptosis of SMCs participated in aortic remodeling [26], we next stained the structure of the abdominal aorta in transverse cross sections. HE staining showed that the thickness of adventitial layers in Ang II-infused mice was reduced following chemerin-9 administration (Figure 4(a)). The EVG results revealed that chemerin-9 prevented complete degradation and deficiency of elastic laminae in the medial layer (Figure 4(b)). Furthermore, IHC staining suggested that chemerin-9 effectively reversed the SMC loss (Figure 4(c)). Therefore, we concluded that chemerin-9 could intervene in AAA pathological progression.

### 3.5. Chemerin-9 Had Anti-Inflammatory and Antiangiogenic Effects on the Experimental AAA Model

**Figure 5:**
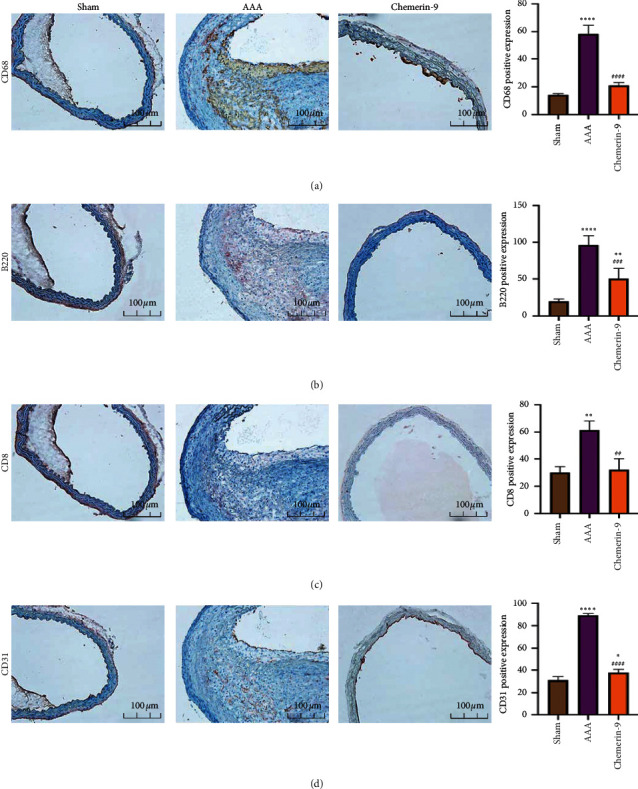
Effects of chemerin-9 on inflammatory cell infiltration and neoangiogenesis in the aortic wall. (a-d) Representative IHC staining images (left) and semiquantitative analysis (right) of CD68, B220, CD8, and CD31 in 3 groups (*n* = 6/group). The data are shown as mean ± SD. ^*∗*^*P* < 0.05; ^*∗∗*^*P* < 0.01; ^*∗∗∗∗*^*P* < 0.0001 vs. the Sham group; ^##^*P* < 0.01; ^###^*P* < 0.001; ^####^*P* < 0.0001 vs. the AAA group.

To further elucidate the pathological changes in the aortic wall mediated by chemerin-9, we performed IHC to detect the marker expression of cells associated with chronic inflammation. Ample infiltration of CD68^+^ macrophages and B220^+^ B cells was found in the media and adventitia in the AAA group (Figures 5(a) and 5(b)), whereas the number of CD8^+^ cells was only slightly increased in the adventitia (Figure 5(c)). Additionally, we found that the AAA mice possessed a higher expression of CD31^+^ microvessels than Sham mice (Figure 5(c)). Intriguingly, these effects were abrogated after chemerin-9 treatment (Figure 5(d)). Collectively, these findings suggested that chemerin-9 might inhibit inflammatory responses, thus lessening the pathological lesions found in AAA.

### 3.6. Chemerin-9 Modulated MMP Expression in Experimental AAA

**Figure 6:**
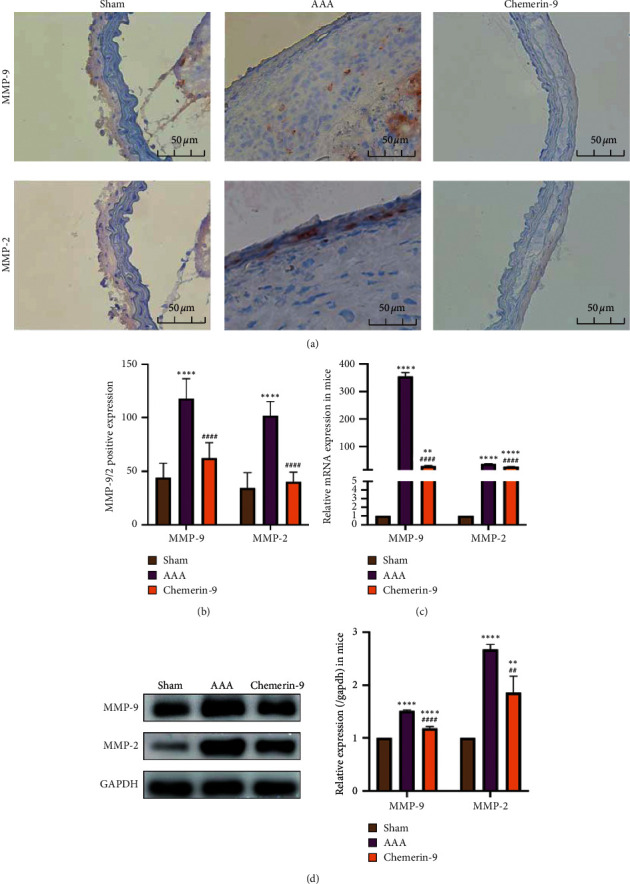
Effects of chemerin-9 on matrix metalloproteinase (MMP)-2 and MMP-9 expression in ApoE^−/−^ mice. (a) Representative IHC staining images of MMP-9 and MMP-2 in 3 groups. (b) Semiquantitative analysis of positive expression of MMP-9 and MMP-2 in 3 groups (*n* = 6/group). (c) Relative mRNA expression of MMP-9 and MMP-2 in the mice aortas (*n* = 6/group). (d) Representative western blot images (left) and semiquantitative analysis (right) of protein expression of MMP-9 and MMP-2 in 3 groups (*n* = 6/group). The data are shown as mean ± SD. ^*∗∗*^*P* < 0.01, ^*∗∗∗∗*^*P* < 0.0001 vs. the Sham group. ^##^*P* < 0.01, ^####^*P* < 0.0001 vs. the AAA group.

Activation of MMPs, especially MMP2 and MMP-9, contributes to the degradation of the ECM and progressive aortic dilation [27]. Next, we explored whether chemerin-9 influenced the levels of MMP2 and MMP-9. As indicated in Figure 6, compared with the Sham group, MMP2 and MMP-9 expression was upregulated in the AAA group, which was partially reversed by chemerin-9. This indicated that chemerin-9 protected the abdominal aorta from MMP damage.

### 3.7. Chemerin-9 Downregulated the Levels of Chemerin and CMKLR1 in Experimental AAA

**Figure 7:**
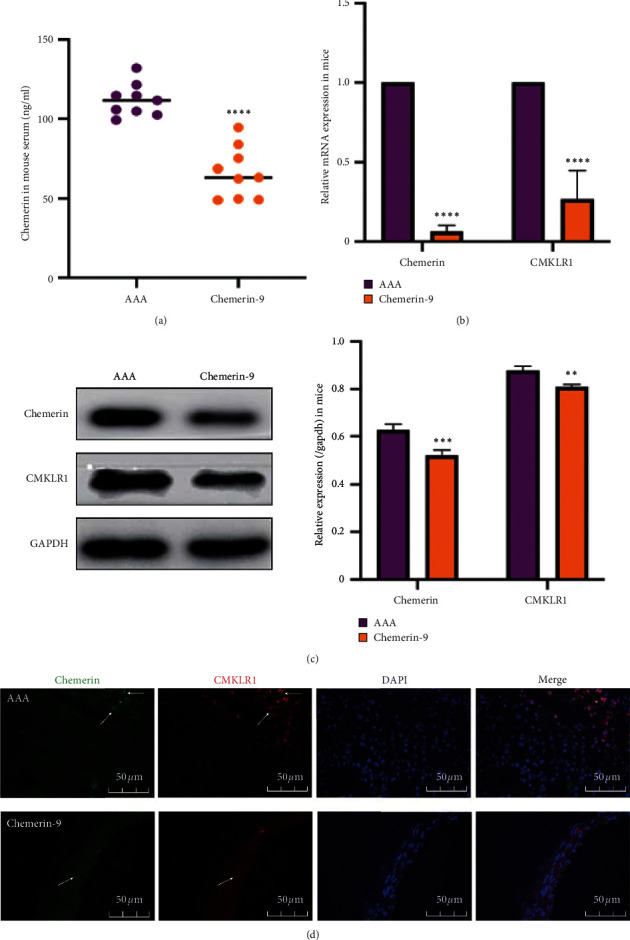
Effects of chemerin-9 on the levels of chemerin and CMKLR1 in ApoE^−/−^ mice. (a) Changes in circulating chemerin levels in mice (*n* = 9/group). (b) Relative mRNA expression of chemerin and CMKLR1 in the aortas of mice (*n* = 6/group). (c) Representative western blot images (left) and semiquantitative analysis (right) of protein expression of chemerin and CMKLR1 in 3 groups (*n* = 6/group). (d) Double immunofluorescence staining for chemerin and CMKLR1 in mice. The data are shown as mean ± SD. ^*∗∗*^*P* < 0.01, ^*∗∗∗*^*P* < 0.001, ^*∗∗∗∗*^*P* < 0.0001 vs. the AAA group.

Simultaneously, we explored the potential mechanisms of chemerin-9 in AAA. Chemerin-9 was infused into the experimental AAA mice, followed by the downregulation of circulating chemerin levels as well as artery-wall chemerin and CMKLR1 expression (Figure 7). Therefore, we speculated that chemerin-9 decreased the levels of chemerin and CMKLR1, thus inhibiting the initiation of inflammatory downstream signaling and eventually decelerating AAA progression.

## 4. Discussion

AAA, progressive structural damage and aortic expansion, is associated with high mortality. AAA is distinguished by elastic fibers and SMC degradation, chronic inflammation, and neovascularization [28, 29]. Loss of SMCs and elastin decreases the strength and compliance of the aortic walls, resulting in the lumen dilation [30–34]. Chronic inflammation also plays an important role in vessel remodeling [35]. MMPs released by macrophages damage elastic fibers and ECM, followed by more inflammatory cell recruitment to the injured site, which forms a vicious circle and eventually leads to either dilatation or rupture of AAA [36–38]. Therefore, it is important to explore novel anti-inflammatory drugs that have been used in clinical AAA therapy [39–41].

Chemerin, a proinflammatory adipokine, is mainly secreted by visceral adipose tissue to regulate the differentiation and metabolism of adipocytes [42]. Elevated levels of circulating chemerin are associated with increased levels of inflammatory markers in several diseases, such as rheumatoid arthritis, psoriasis, and Crohn's disease [43–45]. CMKLR1, a natural receptor of chemerin, is expressed in various inflammatory cells, such as dendritic cells, monocytes, and macrophages [46]. Previous studies reported that the combination of chemerin and CMKLR1 was implicated in cellular migration in response to inflammatory stimuli and was regarded as a prerequisite for macrophage recruitment and endothelial angiogenesis in the cardiovascular system, indicating that there seemed a potential association between chemerin/CMKLR1 axis and AAA progression [47–51]. As expected, the ELISA assay showed that circulating chemerin was elevated in AAA of patients and mice. Compared with the normal aortic tissues, the AAA tissues exhibited higher expression of chemerin and CMKLR1, as revealed by qRT-PCR and western blotting. Therefore, we speculated that chemerin and CMKLR1 might play vital roles in the development of AAA.

Chemerin-9, an analog of chemerin, has a higher affinity for CMKLR1 and functions as an anti-inflammatory regulator to inhibit the development of atherosclerosis, Alzheimer's disease, and pancreatogenic diabetes [22–24]. However, the role of chemerin-9 in the biological behavior of AAA has so far been reported. Herein, we found that chemerin-9 remarkably inhibited the enlargement of AAA diameter and decreased the degradation of elastic fibers as well as SMCs. Furthermore, IHC results showed that the positive percentages of CD8^+^, CD68^+^, B220^+^, and CD31^+^ in the aortic tissues were downregulated following chemerin-9 treatment, suggesting that it inhibited inflammatory cell infiltration and neovascularization. We also discovered that chemerin-9 inhibited MMP-2 and MMP-9 expression, followed by a decrease in ECM degradation. Taken together, these data demonstrated that chemerin-9 could slow down the progression of AAA by regulating the inflammatory response.

Finally, we examined the effects of chemerin-9 on the expression of chemerin and CMKLR1. Results showed that the circulating chemerin and the AAA-tissue chemerin/CMKLR1 expression were downregulated following the chemerin-9 treatment, contradicting previous studies [24, 52]. These conflicts may be attributed to the different dosages or modes of chemerin-9 administration, or the disparate microenvironment of experimental models. Animal models are known to be more reliable than in vitro tests for simulating the inflammatory microenvironment of AAA [53]. The suppression of inflammatory cell accumulation in vivo following chemerin-9 treatment might be an important reason for the decline of CMKLR1 expression. Accumulating evidence suggests that autophagy mediates the inflammatory response to participate in the AAA process [54]. Atg7, an autophagy-related gene, was downregulated by CMKLR1 knockdown, which facilitated the reduction of chemerin level in mice [55, 56]. Therefore, autophagy inhibition may be associated with chemerin decrease mediated by chemerin-9. Nevertheless, the exact mechanisms of chemerin-9 in AAA are warranted to be further investigation. Collectively, our findings suggested that chemerin-9 inhibited the levels of upstream factors chemerin and CMKLR1, thus restraining the activation of the inflammatory response. This may be regarded as a potential mechanism underlying chemerin-9-mediated inhibition of AAA progression.

## 5. Conclusion

We are the first to reveal that the expression levels of chemerin and CMKLR1 were elevated in AAA group, which were partially altered by chemerin-9. Additionally, we demonstrated that chemerin-9 could attenuate AAA progression by regulating the degradation of elastic fibers as well as SMCs, chronic inflammation, and neovascularization, indicating that chemerin-9 has the potential to serve as a novel drug for clinical AAA therapy. However, more in-depth elaboration on the broad regulatory mechanisms of chemerin-9 and their efficacy and safety in a clinical setting is warranted.

## Figures and Tables

**Figure 8:**
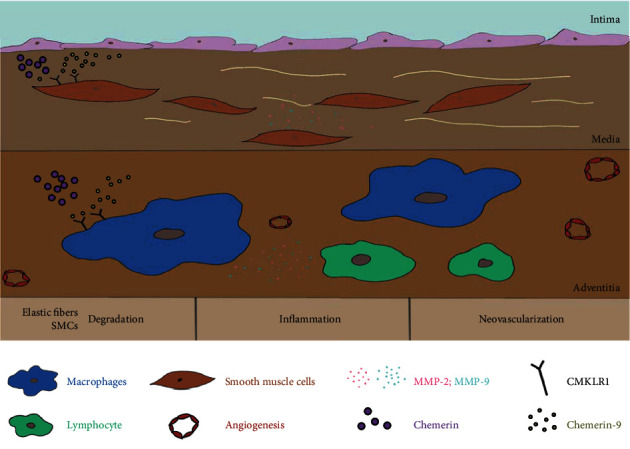
The potential mechanism of chemerin-9 on AAA.

**Table 1:** qRT-PCR primers used in this study.

Gene	Forward sequence	Reverse sequence
Chemerin (human)	GCCCTGGAGGAATTTCACAAGCACC	CACTCGGGTTTCTTCCAGTCCCT
CMKLR1 (human)	GGAACCCCTTTCTTCTAGTGGAC	TCCCAGTTCATGGCAATGCTT
GAPDH (human)	CAGGTGGTCTCCTCTGACTTCA	CACCCTGTTGCTGTAGCCAAAT
Chemerin (mouse)	AATTTAAGCTCCAGCAGACCAAC	ATCCGGCCTAGAATTTTACCCTT
CMKLR1 (mouse)	TGAGGAAGTTACCGCAAACCCAT	GAAGCCCCTCAGGTCCTCGTTC
MMP-9 (mouse)	GCCCTGGAACTCACACGACA	TTGGAAACTCACACGCCAGAAG
MMP-2 (mouse)	GATAACCTGGATGCCGTCGTG	GGTGTGCAGCGATGAAGATGATA
GAPDH (mouse)	TGTGTCCGTCGTGGATCTGA	TTGCTGTTGAAGTCGCAGGAG

## Data Availability

The data used to support the findings of this study are available from the corresponding author upon request.
